# Epigenetic Dysregulation at the Crossroad of Women’s Cancer

**DOI:** 10.3390/cancers11081193

**Published:** 2019-08-16

**Authors:** Rakesh Kumar, Aswathy Mary Paul, Pranela Rameshwar, M. Radhakrishna Pillai

**Affiliations:** 1 Cancer Biology Program, Rajiv Gandhi Centre for Biotechnology, Trivandrum, Kerala 695014, India; 2 Department of Medicine, Division of Hematology-Oncology, Rutgers New Jersey Medical School, Newark, NJ 07103, USA; 3 Department of Human and Molecular Genetics, Virginia Commonwealth University School of Medicine, Richmond, VA 23298, USA; 4 Graduate Degree Program, Manipal Academy of Higher Education, Manipal, Karnataka 576104, India

**Keywords:** women cancer, methylation, acetylation, histones, RNA methylation, epitranscriptomics, chromatin, TET enzymes, receptor tyrosine kinases, estrogen receptor signaling

## Abstract

An increasingly number of women of all age groups are affected by cancer, despite substantial progress in our understanding of cancer pathobiology, the underlying genomic alterations and signaling cascades, and cellular-environmental interactions. Though our understanding of women’s cancer is far more complete than ever before, there is no comprehensive model to explain the reasons behind the increased incidents of certain reproductive cancer among older as well as younger women. It is generally suspected that environmental and life-style factors affecting hormonal and growth control pathways might help account for the rise of women’s cancers in younger age, as well, via epigenetic mechanisms. Epigenetic regulators play an important role in orchestrating an orderly coordination of cellular signals in gene activity in response to upstream signaling and/or epigenetic modifiers present in a dynamic extracellular milieu. Here we will discuss the broad principles of epigenetic regulation of DNA methylation and demethylation, histone acetylation and deacetylation, and RNA methylation in women’s cancers in the context of gene expression, hormonal action, and the EGFR family of cell surface receptor tyrosine kinases. We anticipate that a better understanding of the epigenetics of women’s cancers may provide new regulatory leads and further fuel the development of new epigenetic biomarkers and therapeutic approaches.

## 1. Introduction

Cancer continues to be the second leading cause of mortality globally. Among female neoplasms, breast cancer has the highest incidence and death rate, followed by cervical, endometrial, and ovarian cancers [1,2,3,4]. The potential causes for the growing cancer incidence are multifactorial: the ever-increasing size of the aging population, gene-environment interactions, and a whole range of intrinsic and extrinsic established and suspected cancer risk factors. In addition, even with dedicated global resources for cancer research and treatment, the underlying molecular basis of most, if not all, human cancers remains largely unknown [5]. A poignant example of this is the 2014 World Health Organization (WHO) chart of unknown basis of cancer, which shows that the cause of breast cancers is unknown in 79% of the cases [5]. 

The burden of women’s cancers continues to grow despite substantial progress in our understanding of cancer’s pathobiology, underlying genomic alterations and signaling cascades, and cellular-environmental interactions. A large fraction of gains in extending the life of cancer patients and delaying the recurrence of disease (or sometimes, though rarely, curing the disease) are attributed to targeted cancer therapeutics and evolving combination therapies [6]. One key area of promising treatment is epigenetic therapy which is designed to modify the epigenetic status of the target or targets of interest [7]—the benefits of which are yet to be fully translated and realized into meaningful treatments for cancer common in females. Here we discuss the impact of selective modules of epigenetics, such as DNA and RNA methylation, histone methylation, and histone acetylation, in modifying gene expression in response to upstream signals.

## 2. Changing Trend of Women’s Cancers—Not a Disease of Aging Alone

**Figure 1:**
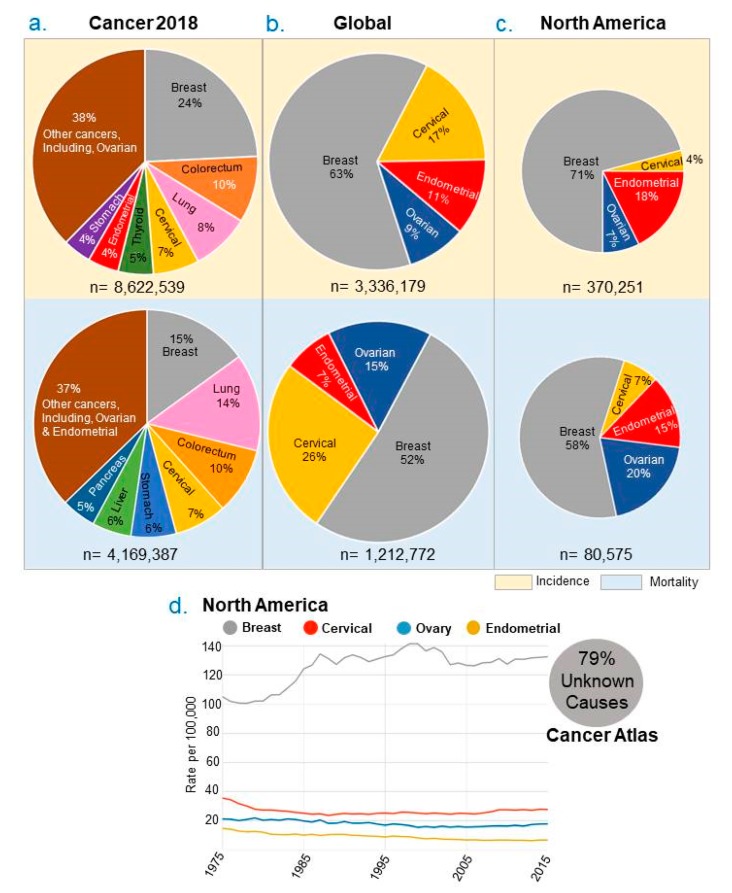
Incidence and mortality of women’s cancer. (**a**,**b**) Global incidence and mortality of breast, cervical, endometrial, and ovarian cancers [1,2,3,4,5]. (**c**,**d**) Incidence and mortality of breast, cervical, endometrial, and ovarian cancer in North America [1,2,3,4,5].

Recently compiled cancer statistics suggest the disproportionately high incidences of new cases of female cancers—breast, ovarian, cervical and endometrial cancer (38.8% of the total global cancer burden in female) and women’s cancer deaths (29.05% of the total global cancer burden). In North America, these numbers are equally as staggering, representing 33.4% of new cases and 24.1% mortality of the total cancer burden (Figure 1a,b) [1,2,3,4,5]. Among female cancers, breast cancer continues to be the most common cancer, globally, including in North America. Following breast, the second and third most common female cancers in North America are endometrial and ovarian cancers, while globally they are endometrial and cervical cancers (Figure 1a–c). More importantly, the average number of new incidences of women’s cancers in the United States also continues to increase for breast cancer (Figure 1d) [1,2,3,4,5]. 

**Figure 2:**
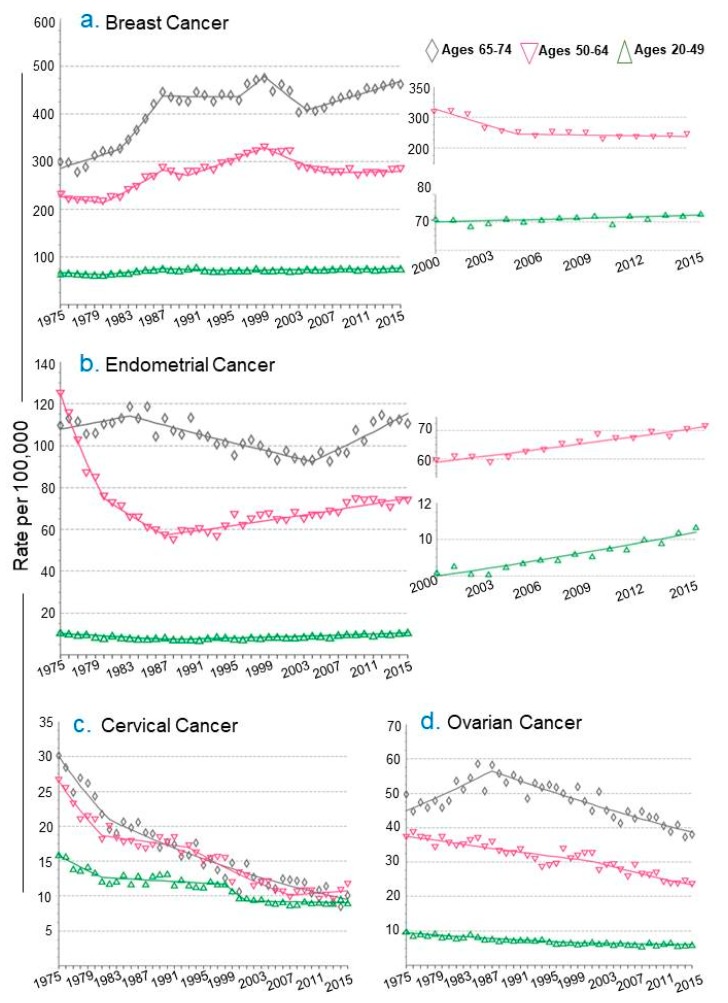
Women’s cancer incidence in North America since 1975. (**a**,**b**) Incidence rates of breast and endometrial cancer as per age-groups. Right inserts, partial enlargements of incidence for two age-groups from 2000–2015. (**c**,**d**) Incidence rates of cervical and ovarian cancers as per age-groups.

A breakdown by age group of new incidences of female cancer indicates that in the 64–74 years age group there is a clear increase of endometrial and breast cancer as compared to cervical and ovarian cancer. The incidence of endometrial cancer shows a distinct increased ratio in the 20–49 and 50–64 year age groups. In the case of breast cancer, there is a clear trend of increased incidences in the 20–49 year age group and in the 50–64 year age group consistently for the past ten years with no evidence of decrease. The modest, but stable, increase in breast cancer in young females will need to be further investigated in the future. (Figure 2a,b). The noted growing trend of endometrial and breast cancer in the 20–49 year age group suggests that these cancers are not just a disease of elderly adults. There is success in the area of cervical cancer and this is partly attributed to the advent of HPV vaccination and prevention programs. The downward trend in ovarian cancer incidence might be due to improved detection and additional treatment options.

**Figure 3:**
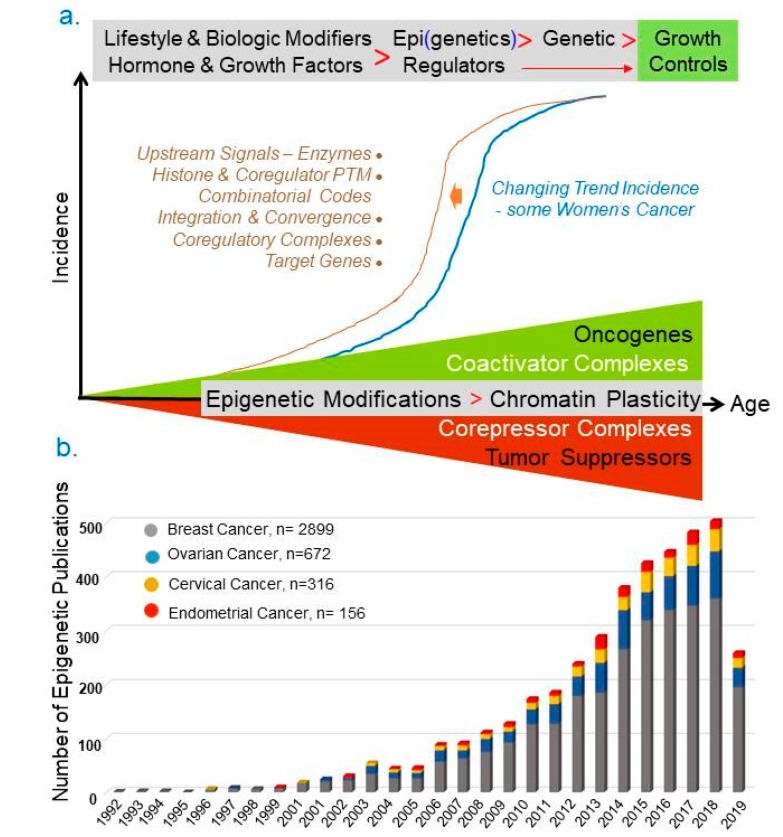
Changing trends in women’s cancer incidence. (**a**) Simplistic view for modifying the growth of cancer cells by epigenetic regulators with or without influencing genetic controls. (**b**) Number of publications for indicated years in PubMed accessed on the 12 May 2019, and searched for “epigenetics” and “epigenetic” with “breast cancer”, “ovarian cancer”, “cervical cancer”, or “endometrial cancer” for indicated years.

The underlying causes behind the overall increase in new incidents of female cancers are complex and remain to be fully clarified. Some of accepted reasons for increased breast and uterine cancer cases include obesity, increased public awareness and diagnosis, and superior screening and diagnostic methods [8,9]. However, these variables are unable to completely account for the noted general trend of increased new cases of breast and endometrial cancers while ovarian and cervical cancer incidences have remained consistent. Because the overwhelming majority of female cancers are hormone-dependent, other recognized risk factors include: age at menarche, nulliparous versus multiparous obstetric history, age at first pregnancy, menopause status, endogenous estrogenic signals, environmental estrogenic signals etc. [10,11,12]. At the moment, there is no comprehensive model to explain the reasons behind the changing trend in female cancers in a manner that could be tested experimentally in a whole animal model. It is generally believed that environmental and life-style factors might constitute the most compelling, modifiable variables in the progression of female cancer via epigenetic mechanisms (Figure 3a). We anticipate that epigenetic regulation of gynaecological cancers in younger women might provide new regulatory leads to help account for the on-going increase in new cases of endometrial and breast women’s cancers, in addition to offering a new layer of mechanistic details and therapeutic approaches for all four types of women’s cancers. 

## 3. Pathobiology of Women’s Cancer

Breast, ovarian and endometrial cancers are hormone sensitive, while the development of cervical cancer is linked with the exposure to human papilloma virus [13,14,15,16]. The process of transformation and maintenance of cancerous phenotypes in female cancers is greatly impacted by ligand- and heterodimerization-induced activation of the EGFR family of receptor-tyrosine kinases (RTKs) and estrogen signaling [6,17,18,19]. In addition, the biology and gene expression machinery are profoundly shaped by the nature of signals generated by regulatory interactions between the tumor cells and tumor microenvironment (TME) and such interactions could be cancer-type specific. The TME is complex, consisting of growth factors, steroids, cytokines, immune cells, fibroblasts, extracellular matrix, and other secretomes such as microvesicles. These factors could cause significant changes in signaling and gene expression machinery [20,21,22,23,24], and ultimately affecting cell functions. 

A large body of work during the last two decades suggests that the process of genomic control of cancer progression is influenced by the coordinated integration of dynamic cellular signals in response to the extracellular milieu via coregulatory complexes and modified histones [25,26]. A closer look into the number of PubMed publications in the area of “epigenetics” and “women cancer” indicate that most of our understanding of epigenetic regulation of female cancers is derived from breast cancer followed by cervical cancer (Figure 3b).

Regulation of gene expression by extracellular signals is profoundly affected by epigenetics—a process of regulating gene expression which could be heritable but independent of changes in the primary DNA nucleotide sequence [25,26,27]. One of the most studied epigenetic modifications is the covalent DNA methylation at the fifth residue in cytosine (5mC) within cytosine-guanine dinucleotide (CpG) islands [28]. Other significant histone posttranslational modifications (PTM) include, acetylation, phosphorylation and methylation [29]. Similar to DNA, mRNAs are also subjected to reversible posttranslational modifications such as N6-methyladenosine (m^6^A) and this area is generally referred to as RNA epitranscriptomic [30,31]. A better understanding of epigenetic controls of gene expression offers an opportunity to advance the on-going development of new epigenetic therapeutic strategies. In recent years, epigenetic cancer therapy is largely directed against modifying two distinct reversible epigenetic marks, namely DNA methylation and histone acetylation, in the context of a set of target genes [32,33,34].

Here we will discuss the broad principles of epigenetic regulation of DNA demethylation and histone acetylation in the context of chromatin remodeling and gene expression, hormonal action, and the EGFR family of cell surface receptor tyrosine kinases. As mRNAs are also modified by reversible PTMs, we delve into the role and dysregulation of enzymes involved in one of the most common PTMs on adenosine, the m^6^A, in female cancers. Due to a vast unevenness in the knowledge available amongst female cancers, we highlight obvious differences in the pathobiology of different female cancers and discuss each type of female cancer. A discussion of the role of RNA-epitranscriptomics will be presented collectively for female cancers due to limited available data and emerging nature of this field. 

## 4. Epigenetic Modules 

There are several layers of epigenetic regulation of the genome, used by external and intracellular milieu, to modify the expression of genes in women’s cancer. We will discuss the role of DNA methylation with a particular focus on demethylation of the 5-carbon position (5mC) in cytosine-guanine (CpG) dinucleotides and histone acetylation in the context of chromatin remodelling and gene expression. We will also discuss the dysregulation of enzymes that regulate adenosine methylation in mRNA at position 6 (hm^6^A) in women’s cancer. 

### 4.1. DNA Methylation

**Figure 4:**
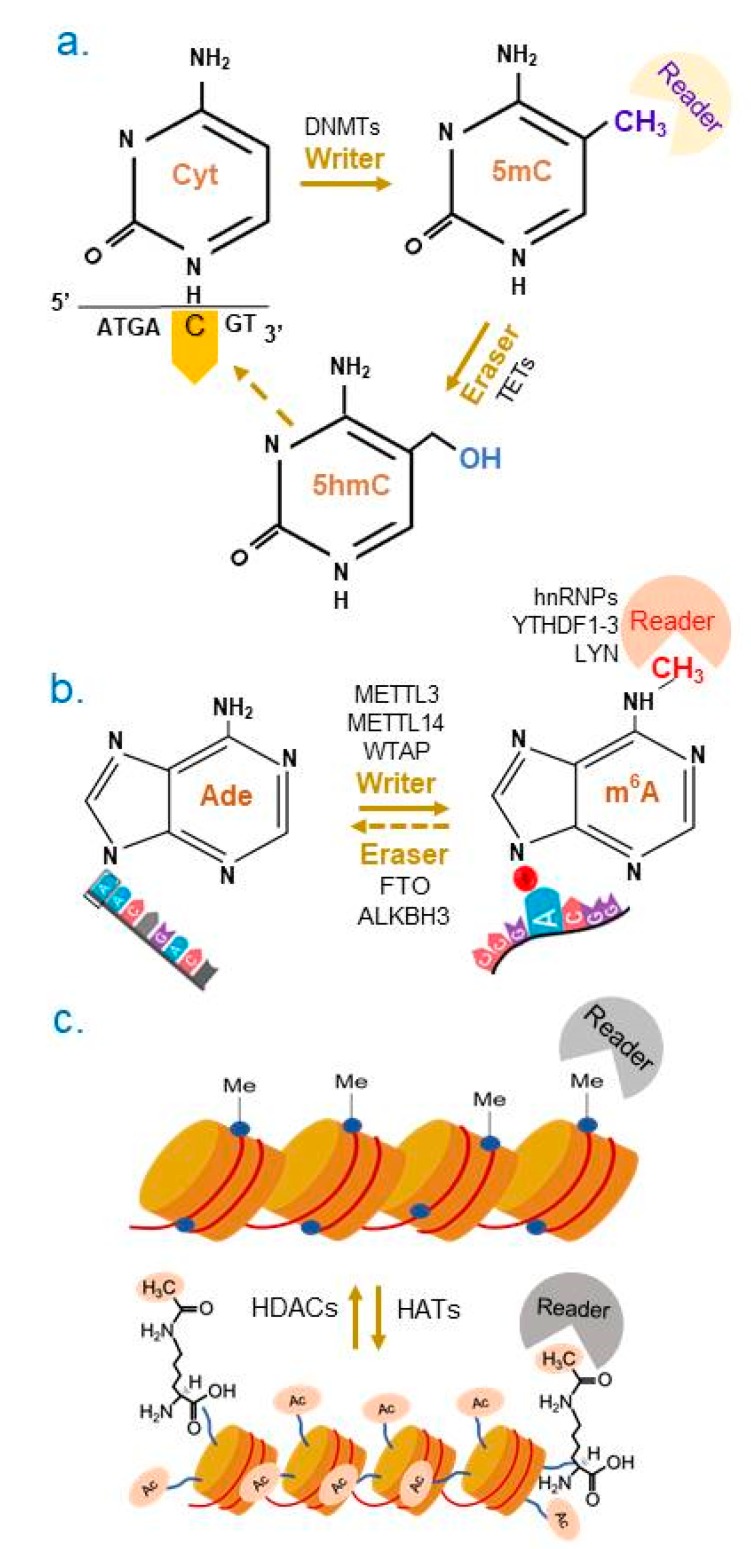
Modules of chemical modifications on cytosine, adenosine, and lysine residues. (**a**) Illustrations showing the DNA methylation at the fifth residue in cytosine (5mC) by DNMTs and demethylation by oxidation of 5mC into 5-hydroxymethylcytosine (5hmC) by TETs, and recycling back to cytosine. (**b**) Illustrations showing the modulation of RNA methylation and demethylation at the sixth position in adenosine, N6-methyladenosine (m^6^A) by writers and erasers, respectively, and recognition of modified base by readers. (**c**) Illustrations showing histone acetylation on ε-N-acetyl lysine and deacetylation by HATs and HDACs, respectively, and recognition of modified base by readers. Ac, acetylation; Me, methylation. Refer to the main test for details.

One of the most common epigenetic modifications is DNA’s cytosine methylation at (5mC) in CpG dinucleotides [28,35] to impart gene silencing (Figure 4a). At the molecular level, methylation of CpG islands is mediated by DNA methyltransferases (DNMTs, such as DNMT1, DNMT3a, and DNMT3b) and in-turn, hypermethylation-triggered transcriptional repression of target genes with CpG islands. 

### 4.2. DNA Demethylation

Equally important for controlling the status of 5-mC is the process that allows oxidation of 5mC into 5-hydroxymethylcytosine (5hmC) (Figure 4a) by ten-eleven translocation methylcytosine (TET) dioxygenase family members, TET1, TET2 and TET3 [36,37,38]. Both the levels and functionality of TET enzymes directly influence the methylation profile of the CpG islands, and hence, modulate associated biological functions. In many cancers, both the expression and enzymatic activity of TETs are also reduced, resulting in increased levels of 5mC and a corresponding decrease in the levels of 5hmC, and increased DNA methylation-linked gene silencing [39]. As opposed to DNMT-mediated 5mC modification, 5hmC is a less well studied epigenetic modification in female cancer and is focused in this review. 

### 4.3. RNA Methylation

Similar to DNA, chemical features and function of mRNAs are modified by reversible posttranslational modifications. Examples of such modifications include, N1-methyladenosine (m^1^A), N6-methyladenosine (m^6^A), N6-hydroxymethyladenosine (hm^6^A), 5-methylcytosine (m^5^C) and 5-hydroxymethylcytidine (hm^5^C) etc. [40,41]. We will discuss the role and dysregulation of enzymes that regulate adenosine methylation in mRNA at position 6 (hm^6^A) (Figure 4b)—one of the most abundant internal RNA modifications—within the consensus motif GAC or AAC in cancer cells. The hm^6^A status of mRNAs regulates pre-mRNA splicing and processing, mRNA export, mRNA decay, cap-independent translation, etc. [42]. 

### 4.4. Histone Acetylation and Deacetylation

Chromatin plasticity plays an important role in allowing transitioning between an open or closed conformation on the target gene chromatin (Figure 4c). This process is carried by a series of regulatory multi-protein complexes that connect DNA-binding proteins with coregulatory complexes and modified histones [26,27,43]. Central to the process of chromatin remodeling is the reversible nature of histone and coregulator PTMs (i.e., acetylation, methylation, or phosphorylation) by enzymes (i.e., histone acetyltransferases, methyltransferases, or kinases) (Figure 4c). Such PTM codes on the histone tails create necessary epitopes for its recognition by chromatin remodeling factors, leading to an orderly formation of chromatin remodeling complexes. In general, histone acetylation of ε-N-acetyl lysine by histone acetyltransferases (HATs) leads to an open chromatin, while histone deacetylation by histone deacetylases (HDACs) to a closed chromatin, and gene stimulation or inhibition [44,45]. 

## 5. Breast Cancer 

Most, if not all, of breast cancer starts as ERα-positive, and this phenotype is progressively lost during cancer progression. Although predisposed mutations in tumor suppressor genes, such as BRCA1, BRCA2 and p53, can lead to breast cancer (as well as other cancer types), an overall low ratio of such cases continues to be a minor causative factor for breast cancer [46,47]. Epigenetic regulation of breast cancer provides an additional layer for integrating extracellular signals into transcriptomic alterations, and acts as a molecular interface to shape the outcome of cross-talk between the ERα and HER family members—two established guideposts for breast cancer biology. Because of signaling-dependent nature of epigenetic PTMs in a given histone, non-histone, coregulator, or mRNA, epigenetics modifications are amicable to potentially correctable therapeutic manipulations for modifying the resulting phenotype. Because epigenetic regulation of breast cancer has been widely covered by several, excellent recent reviews [48,49,50,51,52,53,54], here we will focus on DNA-demethylation after a brief introduction of CpG methylation.

### 5.1. DNA Methylation and Gene Expression

One of the most characterized epigenetic modifications is the hypermethylation of CpG islands in gene promoters by DNMT family of methytransferases [28,35]. The levels of DNMT-1, -3a, and -3b are generally upregulated in sporadic breast cancer and correlates with a poor prognosis; elevated DNMT1 level associates with lymph node metastasis, while DNMT3a and DNMT3b are associated with aggressive stages [55]. The hypermethylation of CpG islands in the promoter region of certain genes also associates with the histone H3K27me3 modification, leading to epigenetic silencing of genes with diverse functions such as transcription, cell-cycle, invasion, DNA repair etc. [56]. Equally important is the genome-wide hypomethylation or demethylation in gene bodies and repetitive sequences in breast cancer, contributing to upregulation of cancer promoting genes such as IL10, MDR1, NAT1, Synuclein and NOTCH1 [57,58,59,60]. The significance of hypermethylation of CpG islands in breast cancer metastasis was further supported by the finding that CpG island methylator pattern involving coordinated hypermethylation of genes correlates with low metastasis risk, while an absence of CpG island methylator generally correlates well with high metastasis [61], suggesting that hypomethylation of oncogenes and hypermethylation of tumor suppressor genes would create favorable milieu during tumorigenesis. Components of epigenetic pathways are also involved in the development of acquired resistance to hormonal therapy in ERα positive breast cancer. For example, increased DNMT status positively correlates with the development of tamoxifen-resistant due to inactivation of ERα, in addition to other pathways [62]; the appearance of activating mutations in the ligand-binding domain of ERα at codons 537 and 538 [63]; increased expression of EHZ2 [64,65]; and a gradual loss of FOXK2 along with the development of ER-/PR-/HER2- phenotypes, etc. [66]. 

### 5.2. DNA Demethylation and Gene Expression

**Figure 5:**
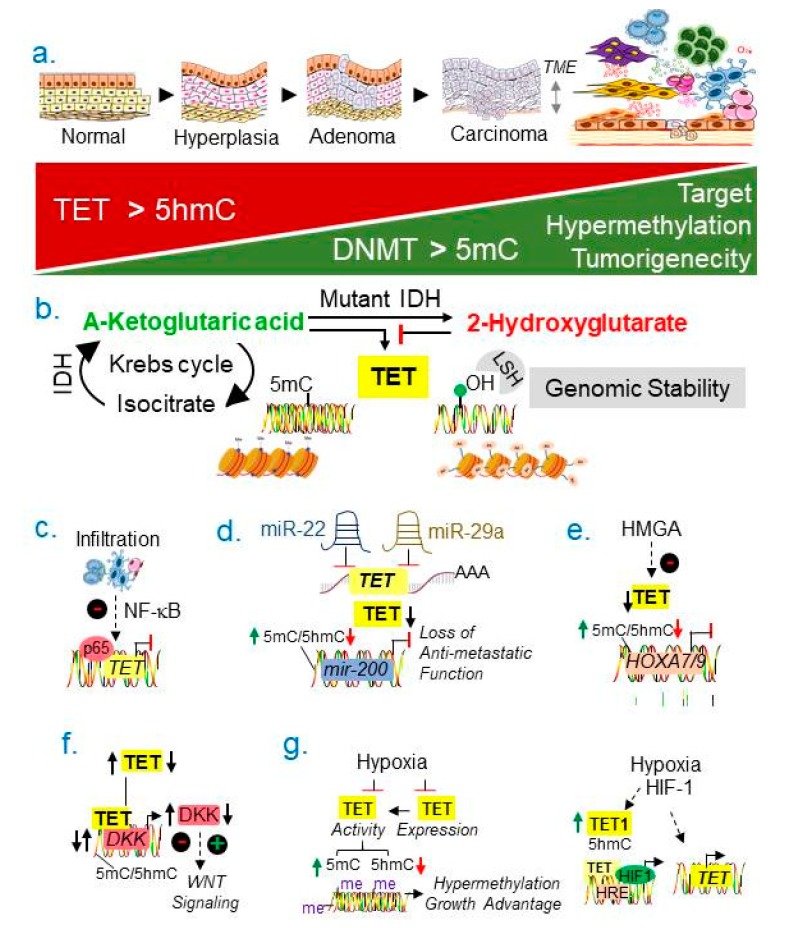
Breast cancer development and DNA-demethylation. (**a**) Illustrations showing breast cancer development and breast cancer—tumor microenvironment (TME) interactions. See Supplementary Figure S1 for description of TME. Reduced levels of TETs and 5hmC and increased levels of DNMT and 5mC in aggressive breast cancer. (**b**) TET activity regulation by ketoglutarate (α-KG) and 2-hydroxyglutarate (2-HG). (**c**) Inflammation regulation of TET activity, (**d**) MicroRNA regulation of TET expression and function. (**e**) HMGA regulation of HOXA7/9 via TET. (**f**) TET regulation of WNT signaling via DKK. (**g**) Left, hypoxia regulation of TET activity and expression; Right, hypoxia regulation of HIF1-alpha-regulated genes.

The levels of 5-methylcytosine (5mC) and consequently, functional outcome of 5mC modification, is tightly regulated by the status of TET1-3 enzymes, in addition to DNMTs [35]. The 5mC is oxidized by TET enzymes into 5 hydroxymethyl-cytosine (5hmC), 5-formylcytosine (5 fC) and 5-carboxylcytosine (5-caC), of which 5hmC is the most predominant oxidized form of 5mC [36,37,38]. In general, downregulation of TETs’ expression as well as a reduction in the level of modified 5hmC during breast cancer progression correlates with a poor prognosis [67] and increased tumorigenecity [36,38,68], presumably, due to increased hypermethylation of putative target genes (Figure 5a). Although TET plays a fundamental role in regulating the levels of methylated DNA, the cellular basis of TET expression and the nature of its upstream modifiers and downstream targets remain poorly understood; and such understanding is just starting to surface. The noted alterations in the levels of functional TETs could be due to its epigenetic silencing, somatic mutations, or upstream activators and repressors. 

The activity of TETs and levels of 5hmC are regulated by cancer-relevant metabolites, commonly called, ‘oncometabolites’. For example, the function of TETs and levels of 5hmC are positively regulated by the level of intracellular α-ketoglutarate (α-KG) generated from the Krebs cycle (Figure 5b), and that α-KG-TET axis participates in supporting the ability of breast tumors to metastasize to the lungs in experimental whole animal setting [69]. Interestingly, α-KG-dependent TET activity and lung metastasis could be effectively blocked by a pharmacological inhibitor of α-ketoglutarate dehydrogenase (KGDH), the enzyme responsible for the generation of α-KG. The presence of mutant isocitrate dehydrogenases (IDH1 and IDH2) and increased accumulation of its metabolite, the 2-hydroxyglutarate (2-HG), are common events in several human tumors, including breast tumor [70]. MYC-overexpressing breast cancer cells exhibit an increased level of 2-HG [71]. Since 2-HG is a competitive inhibitor of α-KG-dependent TET enzymes [70], the balance of α-KG (TET activator) and 2-HT (TET inhibitor) is expected to regulate the activity of TETs (Figure 5b). Functions of several cancer promoting proteins, including, MYC and p53, are positively regulated by their O-linked-β-N-acetylglucosamine (O-GlcNAc) modification by O-GlcNAc transferase (OGT) enzyme [72]. The activity of TET1 in system development is positively regulated by its interaction with OGT [73]. Although OGT is known to be upregulated in breast cancer [74], its relationship with TET1 function in female cancer remains undefined. 

Recent data suggest that *TET1* transcription is repressed by the NF-κB pathway and modified by the immune system in basal-like breast cancer [75]. The noted repression of TET1 expression in breast cancer correlates with NF-κB signaling and infiltration of immune cells in breast cancer; in addition to the recruitment of p65/RelA onto its consensus elements within the *TET1* promoter in breast cancer cells (Figure 5c). 

The expression and function of TETs are also influenced of chromatin remodeling. For example, the expression of TET mRNA is inhibited by pro-cancerous miR-22 and by miR-29a (Figure 5d) [76,77]. Interestingly, miR-22 mediated downregulation of TET expression leads to hypermethylation-linked silencing of the anti-metastatic miR-200 due to inhibition of its promoter demethylation [76]. This suggests a role of miR-network in the regulation of TET expression and functions in cancer cells. Further, breast cancer progression is positively regulated by increased expression of high mobility group AT-hook 2 (HMGA2). 

Chromatin remodeling factor [78], HMGA2 downregulation has been shown to stimulate TET1’s expression in breast cancer cells (Figure 5e). Interestingly, HMGA2-mediated TET1 upregulation could lead to an increased demethylation of the *HOXA7/9* promoter and increased expression of HOXA7/9 [79]. This suggests that under physiological setting, *TET1* expression might be negatively regulated by HMGA2 chromatin remodeling factor, leading to increased methylation and silencing of *HOXA7/9* (Figure 5e)—a step which promotes cancer progression [80]. Interestingly, the expression of EZH2 in triple-negative breast cancer (TNBC) cells downregulate the expression of TET1 via H3K27me3-mediated repression [81]. TET1 overexpression in TNBC cells has been shown to promote hypomethylation, but it stimulates transforming signaling pathways such as PI3K, EGFR and PDGF [82]. 

As breast cancer progression is accompanied by downregulation of TETs as well as upregulation of the WNT pathway, the TET pathway has been also shown to participate in WNT-signaling. For example, TET1 interacts with the *DKK* promoter—an inhibitor of the WNT signaling, inhibits hypermethylation of the *DKK* promoter, leading to increased DKK expression [83], and in-turn, suppression of the WNT signaling. Accordingly, downregulation of TET1—as is the case in breast cancer—stimulates the WNT pathway due to repression of DKK levels (Figure 5f). More recently, the levels of 5hmC appear to regulate the genomic instability via downstream function of 5hmC reader protein, the lymphoid specific helicase (LSH) [84]. Mechanistically, LSH interacts with TET2 and upregulates its expression, increases 5hmC status in the pericentric satellite repeat region, and modulates genome stability. Since breast cancer progression is profoundly affected by TME, hypoxia has been shown to increase genome-wide hypermethylation in experimental breast tumors via inhibiting the activity as well as expression of TETs (Figure 5g) [85]. Such regulatory mechanism controls the expression of genes which provide a growth advantage, and hence, TME could serve a primary driver of the TET activity. Further, hypoxia has been shown to stimulate *TET’*s transcription and levels of 5hmC as well as co-recruitment of TET protein along with HIF-1α onto hypoxia responsive elements in a subset of hypoxia regulated genes in neuroblastoma cells (Figure 5g) [86]. The relevance of these observations in breast cancer remains to be examined. 

The pathobiology of breast cancer development and progression is inherently affected by the HER family and ER/PR receptors. Furthermore, many of the above discussed regulators of TET expression and activity are also expected to be affected by HERs as well as by nuclear receptor signaling. Thus, it would be important to determine the influence of HERs or nuclear receptors on TETs and resulting functions in future studies.

### 5.3. Histone Modifications and Chromatin Remodeling

Epigenetic modifications are integral part of chromatin remodeling and nuclear receptor’s action in breast cancer. Many histone modifying enzymes are component of or interact with the chromatin remodeling complexes that are recruited onto the target gene chromatin in breast cancer cells [25,26]. Two widely studied chromatin remodeling complexes with roles in breast cancer include metastasis-associated protein and SRC families. Interesting, these complexes could either repress or stimulate target gene expression by forming distinct sub-complexes, depending upon the nature of PTMs, in cancer cells [43,87]. Inappropriate ligand-independent activation of histone modifying enzymes promotes cancer progression as well as contributes to the development of hormone resistance [26]. Dysregulation of the chromatin remodeling components could affect the expression of genes with diverse roles in tumor suppression, transformation, DNA repair, cell cycle, metabolism, proliferation etc. [26].

The histone acetylation status on a given genomic loci is under a fine control of HAT-mediated formation of ε-N-acetyl lysine and deacetylation through HDAC-triggered hydrolysis of lysine acetyl moiety. Due to neutralization of the positive charge of histones, its acetylation inhibits electrostatic affinity of the negatively charged DNA and promotes transitioning of heterochromatin to euchromatin. In general, histone H3K9 acetylation and histone methylation on H3K4me1, H3K4me3 and H3K36me3 are linked with an open chromatin and active transcription, while H3K9me3 and H3K27me3 with a closed chromatin and gene repression. Further, both active and repressed histone methylation could coexist on the promoters of certain genes. 

In addition to epigenetic alterations on specific target genes, dysregulated dynamic changes in the status of epigenetic histone modifications reflect the genome wide chromatin remodeling, allowing certain genes to be repressed, derepressed, activated, or modify from their poised state. For example, the observed reduction in the levels of active transcription marks such as H4K16-acetyl or H4K12-acetyl in the early stages of breast cancer, ductal carcinoma in-situ, or ductal carcinoma suggests that these changes may lead to reduced levels of target genes that might not be involved in active proliferation [88]. Transformation of breast cancer cells has been shown to be also accompanied by a reduction in the genome-wide levels of histones H3K9me2 and H3K9me3 (suggestive marks for derepression of target genes) and an increased H3K4me3 (a mark of active promoter) in TNBC cell lines [89], implying increased expression of putative target genes.

The transcription of ERα is stimulated by MYST3 in a HAT-activity dependent manner. Since MYST3 is overexpressed along with ERα in breast tumor, it is possible that MYST3 might be also involved in a ligand-independent transactivation of ERα, to impart to hormone resistance [90]. As histones are subjected to undergo multiple epigenetic PTMs in a dynamic manner in breast cancer cells, it is expected that the net transcription outcome is influenced by the cumulative effect of transregulation of PTMs in histone as well as in coregulators. For example, G9a methytransferase acts as a coactivator for ERα transactivation activity in breast cancer cells by dimethylating K235 in ERα, and in-turn, ERα-K235me2 promotes the formation of the PHF20/HAT complex onto the ERα target gene promoters [91]. Similarly, SIRT1 appears to control the levels of acetylation of histone H3K4, H3K9 and H4K16 marks on the target gene promoters in breast cancer cells. Further, the nature of ER-regulated genes is profoundly regulated by coactivators and corepressors, such as SRCs and MTAs, respectively [25,26]. In this context, ASXL2—a newly identified ERα coregulator—supports ERα transactivating function via regulating the status of methylation on histone H3, K4, K9, and K27 on the ER-target genes in breast cancer cells [92]. In addition to histone PTMs, increased expression of methyl-CpG binding protein 2 (MBP2), which recognizes methylated CpG islands, correlates with the status of H3Ac and H4Ac modifications in breast invasive ductal carcinoma, suggesting a widespread alteration in gene expression via methylation- and acetylation-pathways [93]. 

As epigenetics of breast cancer is immensely influenced by TME signals, the nature of transcriptomic alterations in breast cancer cells is also regulated by the ligands for the HER family members as well as by hypoxia-mediated enhanced stability of G9a methytransferase [94]. Like-wise, immune cells—another major component of TME, could upregulate the expression of PD-L1 expression in cancer stem cells and modify the nature of interactions between the TME and breast cancer cells [95]. In this context, recent studies suggest that EMT associated upregulation of PD-L1 in cancer cells might be regulated in a context-dependent manner—as there was an increased recruitment of an active transcription histone mark (H3K4me3), at the expense of repressive histone marks (H3K9me3, H3K27me3), on the *PD-L1* promoter in tumorspheres but not monolayer breast cancer cells [96]. In addition, it is possible that epigenetic pathways not only contribute to the effect of TME on the biology of cancer cells, but also influence gene expression in the tumor microenvironment. For example, polarization of tumor-associated macrophages (TAMs, a major component of TME) into the M1 or M2 phase could be modulated by regulating the activities of epigenetic enzymes [97]. This is an emerging research area with no experimental data for breast cancer. Because M1 and M2 macrophages participate in inhibiting and supporting the tumor growth, respectively, the ideal epigenetic inhibitor would be the one which could either inhibit the tumor supporting M2 phenotypes or promote the transitioning of the M2 phenotype into M1 phenotype or contribute to both of these functions. 

## 6. Ovarian Cancer

Among ovarian cancers, the cell surface derived epithelial ovarian cancer (EOC) is the most common disease, accounting for about 75–90% of all ovarian cancer types. The EOC consists of commonly diagnosed high grade serous ovarian cancer (HGS), low grade serous (LGS) and mucinous (MOC), endometrioid, and clear cell carcinomas sub-types. The EOC subtypes exhibit characteristics genomic features: HGS which starts from the fallopian tube or the surface epithelium generally shows chromosomal instability [98], while LGS sub-type exhibits chromosomal stability combined with mutations in the RAS pathway which, in-principle, could drive the process of oncogenesis over a period of time [99]. Recent studies suggest that epigenetic reprogramming might be an early event in the case of high grade serous-type with BRCA1/2 mutations (and derived from fimbrial cells). This is evident in specimens with BRCA mutations where there is finding of substantial methylation of CpGs in the fimbrial region, but not the proximal region, of the fallopian tube [100]. Similarly, loss of an E3 ligase RNF20 in the fallopian tube epithelium-derived cell lines has been shown to reduce the level of histone H2B monoubiquitylation. In-turn, this promotes a relaxed chromatin conformation as well as increased expression of immune regulators [101]. 

As opposed to other female cancer, ovarian cancer is generally diagnosed at late stages when the disease has already disseminated to the peritoneal cavity. Once detached from the primary sites, ovarian tumor cells undergo anoikis-associated cell death. However, exfoliated ovarian cells soon become anoikis-resistant and start populating in the peritoneal space as clusters, adhering to other organs in the peritoneal cavity. Because peritoneal region is rich in growth factors, cytokines and soluble factors, immune and other cell-types, peritoneal region provides a nurturing microenvironment for supporting the proliferative and survival signaling in ovarian cancer cells [102,103]. 

**Figure 6:**
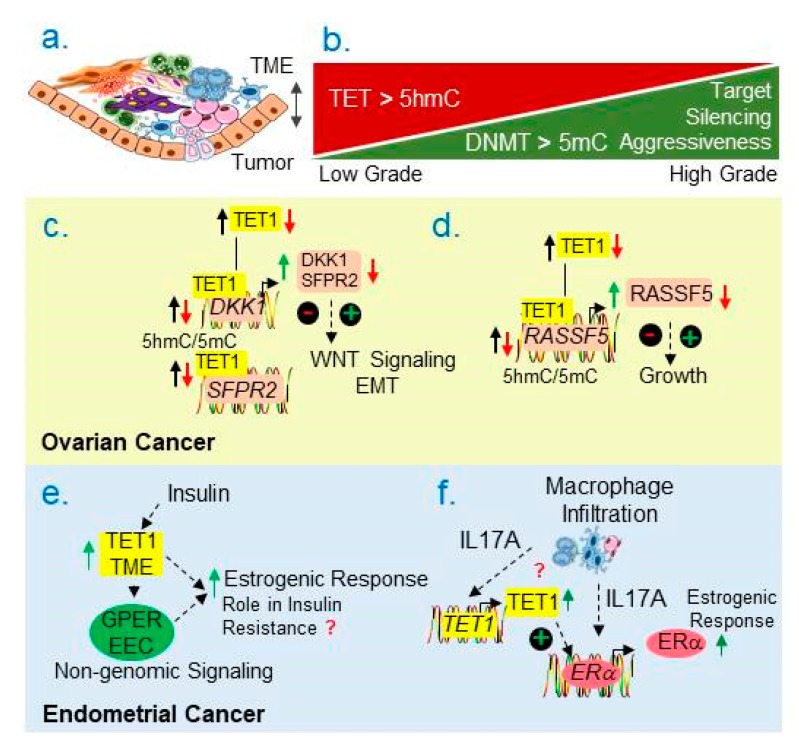
DNA 5mC status and cancer aggressiveness. (**a**) Illustrations showing the tumor and tumor microenvironment interactions (TME). See Supplementary Figure S1 for description of TME. (**b**) Reduced levels of TETs and 5hmC and increased levels of DNMT and 5mC in aggressive ovarian and endometrial cancers. (**c**) TET regulation of WNT signaling via controlling the transcription of negative regulators DKK and SFPR2. (**d**) TET1 regulation of ovarian cancer growth via tumor suppressor RASSF5 expression. (**e**) Insulin stimulation of TET1 expression in TME cells and upregulation of non-genomic GPER signaling in endometrial cancer cells. (**f**) Macrophage infiltration linked released of IL17A cytokine in re-expression of ER in endometrial cancer cells via modulating the level of TET1 expression.

Most, if not all, patients with ovarian cancer exhibit a recurrence of the disease and those who initially respond to therapies also develops resistance to the first line of platinum- or taxane-based chemotherapies. Although patients with a defective *Brca1* respond to the poly (adenosine diphosphate [ADP]-ribose) polymerase (PARP) inhibitors but exhibited very little or no significant gains in an overall survival of patients [104], highlighting the need to further study the role of epigenetics in understanding and treatment of ovarian cancer. The biology of ovarian cancer progression and its ability to acquire therapeutic resistance are also affected by the nature of cellular and biochemical interactions between the tumor cells and TME in the intraperitoneal cavity (Figure 6a). Such cellular interactions result in dynamic changes in gene expression in response to persistent or dynamic changes extracellular milieu via epigenetic machinery.

### 6.1. DNA Methylation and Gene Expression 

As CpG dinucleotides are distributed throughout the genome, hypermethylation of CpG islands in the target promoter regions lead to the loss or silencing of tumor suppressors during cancer progression [105,106,107]. In this context, the status of TET, 5hmC, DNA hyomethylation, and CpG hypermethylation of tumor suppressors are common events in women’s cancer (Figure 6b). Examples of commonly hypermethylated genes in ovarian cancer include: BRAC1, PTEN, HIC1, E-cadherin, APC, MLH1, HIC1 etc. [108,109,110], cell adhesion genes such as ICAM-1, apoptosis genes such as PAR-4, cell cycle inhibitors p16, TUBB3 [111,112,113,114]. There are also examples of cell-type specific gene silencing in ovarian cancer cells. For example, tumor suppressor Ras association domain family member 1 (RASSF1A) and O6-methylguanine DNA methyltransferase (MGMT) are lost in invasive ovarian cancer [115], while Multiple Sclerosis (MS1) and Wilm’s tumor (WT1) are largely lost in endometroid ovarian cancer but not in serous ovarian cancer [116]. These differences might be due to the involvement of cell-type specific regulatory factors. Interestingly, ovarian cancer progression is also accompanied by increased expression of enzymes responsible for DNA methylation, DNMT1 and DNMT3a, and expression levels correlate well with a poor prognosis of patients with ovarian cancer [117]. Equally important to the biology of ovarian cancer is the region of hypomethylation of CpG repetitive sequences which are generally associated with the genomic instability. In addition, DNA hypomethylation could upregulate the expression of cancer promoting genes such as maspin, CLDN4, brother of the regulator of imprinted sites (BORIS) and HOXA10 [118,119,120,121,122]. This raises an obvious concern about the possibility of activating oncogenes by demethylating agents. In addition, it remains unclear how the process of preferential methylation of CpG islands over CpG repetitive sequences is achieved by DNMTs and the role played by the chromatin remodeling factors in a context-specific architecture of the nucleosome.

### 6.2. Emerging Role of DNA 5mC Demethylation 

In general, TET1 expression is reduced in invasive ovarian cancer as compared to less invasive stages [123]. Consistent with this observation, a global reduction in the level of 5hmC in high-grade serous ovarian cancer associates with a poor survival of ovarian cancer patients [124]. Interestingly, a global reduction in the level of 5hmC could be revered using epigenetic approaches involving DNMT inhibitors [124] and thus, supports the notion of TET1’s involvement in reprogramming ovarian cancer epigenome [125]. Experimental restoration of TET1 repression in ovarian cancer cells could also inhibit the growth of ovarian cancer cells by reversing DNA-methylation-associated silencing of *SFPR2* and *DKK1*—two endogenous inhibitors of WNT signaling [123]. This, in turn, suppresses both Wnt signaling as well as associated EMT (Figure 6c). In some cases, TET1 upregulation was also reported in aggressive ovarian cancer; however, the basis of such overexpression and it’s implication in patient survival through specific pathways remains undefined [125]. In general, it is believed that excessive DNA demethylation by TET1 overexpression might lead to hypo-methylation linked activation of growth promoting genes and oncogenes. One of the major targets of TET1 is *RASSF5*, a tumor suppressor—as TET1 overexpression upregulates RASSF5 expression by inhibiting the hypermethylation of the *RASSF5* promoter (Figure 6d) [126,127]. These observations suggest that to better appreciate the relationship between the DNA methylation and demethylation, it will be important to evaluate the levels and activities of DNMTs and TETs as well as the status of 5mC and 5hmC in the same set of clinical specimens. Establishing a physiological relevance of TETs will be essential before formulating testable mechanistic studies as well as for examining the outcome of DNMT-directed experimental approaches.

### 6.3. Histone Methylation and Chromatin Remodeling

Among methyltransferases in ovarian cancer, the Enhancer of Zeste 2 (EZH2)—also known as Polycomb Repressive Complex 2 (PRC2)—has emerged as an important epigenetic regulator of ovarian cancer biology. The levels of EZH2 are significantly upregulated in ovarian cancer and correlate with a poor prognosis of ovarian cancer patients [128]. As a part of EZH2/SUZ12/EED corepressor complex, recruitment of EZH2 on the target gene chromatin supports transcriptional repression [128]. In addition to histone methylation-associated inhibition of target gene transcription, EZH2 is unique as it binds to DNMT as a part of PCR2/EZH2 complex, and gets recruited onto EZH2’s target genes for facilitating DNA-methylation [129]. EZH2, which is commonly upregulated and overexpressed in invasive cancers, could promote a pro-tumorigenic phenotype by functioning as a negative regulator of proliferation. Accordingly, experimental targeting of EZH2 triggers the cell death and inhibits the cellular invasion [130] as well as inhibits the expression of target tumor suppressor gene, such as transforming growth factor-beta1 (TGF-β1) [131]. In certain ovarian cancer cells, the noted growth inhibitory effects of EHZ2 inhibition are attributed to increased levels of phosphoinositide-3-kinase interacting protein 1(PIK3IP1) which antagonizes the cell survival signaling [132]. 

The levels of EZH2 activity on the target chromatin are positively regulated by its interactions with other components of the PRC2 complex as well as by its phosphorylation on T350 by cyclin-dependent kinases or on S21 by AKT [133,134]. As EOC contains hyperactivated PI3K-AKT pathway and the fact that AKT is a target of EGFR/HER2 and non-genomic estrogen signaling [135], it will be important to develop an integrated view of pathways converging onto EZH2. At present, the nature of signals which might be contributing to increased EZH2 expression in ovarian cancer remains unknown. The effectiveness of a given epigenetic targeting agent is likely to be further dependent on an overall epigenetic signature of a given cell-type. For example, EZH2 inhibitor inhibits the growth of ovarian cancer cells which are positive for arginine methyltransferase PRMT4/CARM1, which itself is upregulated in ovarian cancer [136]. EHZ2 inhibition efficiently inhibits the growth of ovarian cancer cells which contain mutated ARID1A [136]. As ARID1A is mutated in about one-half of clear cell ovarian carcinomas, the above observations suggest that such patients could be further stratified on the basis of various components of EZH2 pathways to maximize the therapeutic effects of EZH2 inhibitors. 

Components of the chromatin remodeling complexes, such as ARID1A (BAF250A) of the SWI/SNF complex and Rsf-1 of the ISWI complex, are mutated and/or overexpressed in ovarian cancer, respectively [137,138,139]. In addition, ARID1A cooperates with other mutated epigenetic or oncogenic signaling such as mutated PI3KCA in ovarian cancer [140]. Studies from mouse models suggest that mutant ARID1A alone does not promote ovarian tumorigenesis but co-occurrence of mutated ARID1A and PI3KCA might be needed for a prolong upregulation of pro-inflammatory IL6 [141]. Because ovarian cancer cell grow in the peritoneal cavity, secreted IL6 is likely to initiate its own cascades of oncogenic pathways, further highlighting the significance of ovarian cancer-TME interactions. This suggest that co-occurrence of different layers of regulatory networks could be considered for formulating novel prognostic approaches to utilize a battery of biomarkers taking into consideration. As epigenetic status of its nucleosomes is an important determinant of gene transcription, gaining additional mechanistic insights might offer new clues about the regulation of ovarian cancer progression by epigenetic pathways. 

Another histone methyltransferase with an emerging role in ovarian cancer is DOT1-like protein (Dot1L) which lacks the conversed SET domain and responsible for histone H3 methylation on lysine 79. DOT1L is widely overexpressed in ovarian cancer and stimulates cell cycle progression via stimulating the *CDK6* transcription [142]. The DOT1L upregulation in ovarian cancer cells might also contribute to drug resistance as it gets recruited onto the promoters of drug-resistant genes via transcriptional factor C/EBPβ. This, in-turn, regulates the expression of genes with roles in platinum resistance [143]. Ovarian cancer cells also contain easily detectable levels of the protein arginine methyltransferases (PRMTs), which adds a methyl group to arginine in histone H3 and inhibits the expression of tumor suppressors. PRMT5 overexpression correlates well with increased proliferation and disease progression [144,145]. 

In addition to histone methyltransferases, the steady state levels of methylation of histones as well as non-histone proteins are affected by demethylases, such as Lysine-specific demethylase 1 (LSD1) which demethylates H3K4 and K9 marks on the target gene chromatin. The LSD1 is widely overexpressed in ovarian cancer and associates with a poor prognosis [146,147]. Interestingly, LSD1 is a context-specific epigenetic regulator as it inactivates *E-cadherin* transcription owing to H3K4me2 demethylation, but stimulates ERα transactivation activity via directly interacting with it [148]. Increased levels of LSD1 in ovarian cancer cells associate with invasion and expression of EMT markers [149].

### 6.4. Histone Acetylation and Chromatin Remodeling 

Although there have been a number of large scale transcriptomic studies in ovarian cancer [150], the role of lysine acetylation and deacetylation of histones by HATs and HDACs, respectively, continues to be understudied. It appears that the levels of human males absent on the first (hMOF)—a HAT responsible for acetylation of lysine 16 in H4, are downregulated in ovarian cancers as compared to normal ovarian tissues [151,152]. Although, hMOF downregulation has been shown to be associated with the genomic instability and dysregulated DNA damage response [153], its role in ovarian cancer remains unclear. In general, class I HDAC1-3 are upregulated during ovarian cancer progression and correlate with a poor patient survival [154,155]; while class II HDAC4 overexpression is linked with platinum resistance as well deacetylation of STAT1 in primary ovarian cancer cells isolated from ovarian cancer patients [156]. Among the class III Sirts, Sirt1 is upregulated in chemoresistant EOC [157]; acquired cisplatin resistance associates with alterations in the expression of BRCA1, SIRT1 and EGFR in ovarian cancer cells [158]; SIRT3 and SIRT6 are downregulated in ovarian carcinoma [159,160]; and SIRT4 overexpression in ovarian cancer cells modulates invasiveness [161,162].

### 6.5. HER Family and Chromatin Remodeling 

Ovarian cancers express easily detectable or overexpressed levels of HERs. The founding member of the family, the EGFR, is widely overexpressed in ovarian cancer [163,164,165]; while HER2 is overexpressed in a subset of ovarian mucinous tumors [166,167]. There are also examples of upregulation of HER2 mRNA, in the absence of HER2 overexpression in ovarian clear cell carcinomas (OCCCs) [168]. Because overexpression of EGFR or HER2 is not a prerequisite for a hyperactivated HER signaling, HER2-signaling is primarily hyperstimulated by TGFα or heregulin-induced EGFR/HER2 and HER3/HER2 dimers [17,18,19,169], one can’t rule out the possibility of an active HER2 signaling in ovarian cancer, even in the absence of HER2 upregulation. In addition to HER family members, ovarian cancers also overexpress c-MET receptor [170], raising a possibility of engaging c-MET/HER dimerization in a sub-group of ovarian cancer. It is noteworthy to mention that the peritoneal cavity contains a variety of secreted polypeptide factors which could potentially stimulate EGFR and HER3 signaling in tumor cells, and contribute to a prolonged mitogenic signaling [171]. For example, engagement of EGFR and HER3 signaling by HB-EGF and heregulin, respectively, activates YAP1 transcription regulator which in-turn, increases the expression of EGFR and HER3 as well as several of their ligands in ovarian cancer cells [172]. Such autocrine loops might provide good working models to tease-out, various regulatory and feedback layers of epigenetic control of gene expression during ovarian cancer progression. 

In addition to metastasis into the peritoneal cavity, most of patients with advanced ovarian cancer also exhibit metastasis to the omentum [173]. This suggests an inherent role of stromal fibroblasts and secreted growth factors in omental microenvironment for promoting ovarian cancer metastasis from the primary sites. In this context, paracrine heregulin present has been shown to stimulate HER3 signaling in ovarian tumor cells and drive the process of omental metastasis and this process could be interrupted by selectively targeting HER3 [173]. Similarly, there are also examples of increased expression of TGFα in stromal fibroblasts by secreted TNFα from cancer cells and in-turn, stimulation of EGFR signaling in ovarian cancer cells by TGFα secreted from omental fibroblasts [174]. In brief, ovarian cancer progression is profoundly regulated by the nature of signals and TME interactions via HER-dependent transcription of target genes. However, the contribution of epigenetic machinery in the HER-driven pathogenesis of ovarian cancer remains poorly understood.

There is some evidence to suggest a role of EGFR signaling in upregulating DNMT activity and DNA methylation in ovarian cancer cells [175]. EGFR signaling stimulates the expression of LSD1 in ovarian cancer cells, and EGFR-LSD1 pathway plays a mechanistic role in the cell migratory activity of EGFR, presumably, via modifying the methylation status of its putative targets [176]. It remains unclear if the functionality of mutated and/or overexpressed epigenetic regulators would be augmented or antagonized by activated HER signaling in ovarian cancer. The above observations raise the possibility of regulation of epigenetic machinery by HER signaling and such work might provide a rationale for combination therapy involving HER-directed and -epigenetic inhibitors.

### 6.6. Estrogen Signaling in Epigenetic Regulation of Ovarian Cancer 

In addition to understudied role of HERs in EOCs, we do not have a full appreciation of the role of hormone signaling in epigenetic regulation of ovarian cancer progression. Although clear cell carcinoma expresses low level of ERα, the EAC and serous high grade carcinomas express an easily detectable level of ER ERα-implying an active estrogenic signaling [177,178,179,180]. During ovarian cancer progression, the levels of ERα are generally silenced due to its hypermethylation. It’s possible that the loss of ERα expression could reset the progression path to the development of ERα negative aggressive tumors from ERα positive tumors—similar to breast cancer. However, the issue of estrogen signaling in ovarian cancer might be best examined in the context of subcellular localization of ERα and ERβ in the nucleus and the cytoplasm, resulting in genomic and non-genomic signaling, respectively [181,182]. Although the levels of ERβ are lost with progression [183], the status of the nuclear and cytoplasmic ERβ differentially correlates with ovarian cancer progression markers [184,185,186]. As a substantial amount of ERβ resides in the cytoplasm, cytoplasmic ERβ could potentially participate in non-genomic signaling; however, this aspect of signaling has not been examined. It remains possible that EGFR/HER2 signaling may not only antagonize growth inhibitory functions of ERβ signaling, but also feed into epigenetic inactivation of ERα. At the moment, there is not much information whether the status and activities of epigenetic enzymes might be influenced by the non-genomic component of ER signaling and by RTK-ER axis in ovarian cancer. Further studies are needed to fill-up these representative gaps for gaining a comprehensive portrait of epigenetic regulation of ovarian cancer.

## 7. Endometrial Cancer

Although endometrial cancer is generally considered a postmenopausal disease, a subset of patients continues to be younger for reasons which are not fully understood. Some of the risk factors for type I endometrial cancers are: changes in the levels of estrogen and progesterone, nature of unopposed estrogen treatment, early menarche, nulliparity, xenoestrogens, obesity, use of tamoxifen therapy for breast cancer, and environmental factors [187,188]. Among endometrial cancer, high grade Type II poorly differentiated cancers have a tendency to invade, express low ERα; while low grade Type I well-differentiated adenocarcinomas in pre-menopausal patients express ERα with an active signaling [189,190]. In general, type II endometrial carcinomas, including, the uterine papillary serous carcinoma (UPSC) are high grade, and accounts for the majority of mortality in patients with endometrial cancer [191]. The development of endometrial cancer is likely to be also regulated by extrinsic environmental and life-style factors via cellular epigenetic machinery. The epigenetic regulation of endometrial cancer is an exciting area of research, but still at an early stage. Here we illustrate selected examples of epigenetic regulation of endometrial cancer cells and bring out the pockets of new discoveries in the field.

### 7.1. Hormonal Regulation of Endometrial Cancer

The normal endometrium contains receptors for estrogen and progesterone and responds to changing dynamic in the levels of steroid hormones with appropriate physiological responses. In principle, estrogen-mediated proliferative signaling is counteracted by antiestrogenic effects of progesterone receptor B (PR-B), such as suppression of ER’s expression and estradiol metabolism. The proliferation of uterine epithelium is regulated by interaction between the growth stimulatory ERα and growth inhibitory PR signaling [187]. A potential dysregulation of this cascade and underlying molecular regulatory steps could re-set the endometrial homeostasis in favor of a hyperproliferative response [192,193], and eventually, lead to cancer over a period of time. The current therapeutic options are focused on restoring the growth inhibitory PR signaling by appropriate hormonal therapy. However, about one-third of patients with well-differentiated cancer resist the benefits of hormonal therapy [194], in part because of reduced expression or activity of PR-B [195]. 

The balance of proliferative responses in endometrial is influenced by the growth inhibitory signals such as PR and stimulatory signals such as MYC. The loss of PR expression and functionality in endometrial cancer cells could stimulate MYC the expression of MYC [196]. In addition to an imbalance in the levels of ER/PR signaling and their effector nuclear receptor (NR) coregulators [197], the development of endometrial cancer is also regulated by the DNA repair activity of DNA mismatch repair (MMR) pathways, silencing/ mutations in tumor suppressors, and dysregulation of the chromatin remodeling pathways [198,199,200,201]. In general, a reduction in the level of MMT repair activity combined with growth promoting ER’s genomic- and -nongenomic signaling events might also contribute to growth stimulation. Because about 25–40% of endometrial cancer contains MMS mutations or methylation-linked silencing [202], the role of epigenetic in the regulation of endometrial cancer remains wide-open. 

The process of cancer progression is profoundly under the control of regulatory interactions between the tumor and TME (Figure 6a) [203]. Examples of the involvement of TME components in endometrial cancer include: hormones, pro-inflammatory cytokines and growth factors, marcophages and fibroblasts as well as the status of hypoxia and pH etc. The significance of TME in endometrial cancer is underscored by an observation showing that genetic inactivation of PR in stroma—due to PR’s hypermerhylation—in a PTEN knockout mouse model of endometrial cancer could antagonize PR’s growth inhibitory activity in endometrium [204]. 

As pathogenesis of endometrial cancer is driven by an imbalance of ER and PR signaling in the context of circulating estrogen, external phytoestrogens and endocrine disruptors and their metabolites [187], one of less studied research area includes examining the effects of dysregulated hormonal signaling originating from the wild-type ERs and/or its variants on the functionality of the chromatin remodeling machinery in endometrial cancer. This might be particularly important for two reasons: chromatin remodeling pathways play a mandatory role in executing transcriptional effects of ER and PR, and the fact that environmental and certain phytoestrogens factors are thought to act as modifier of stimulatory effects of hormonal signaling in the endometrial cancer. In addition, the biology of certain sub-types of endometrial cancer is likely to be affected by the nature of cancer cell-TME interactions as well as cross-regulation of ER/PR and HER signaling by growth factors and cytokines present in TME. Similarly, components of chromatin remodeling pathways such as MBD proteins have been shown to be recruited on the *PR-B* gene and associated with its methylation-linked silencing. However, we don’t know what kind of upstream signals trigger the recruitment of such corepressive complexes onto the *PR-B* gene chromatin.

In addition to steroid signaling, epigenetic regulation of endometrial cancer is likely to be mechanistically influenced by pathways triggered by the HER family of the cell surface receptor tyrosine kinases. Previous reports suggest that the HER signaling regulates gene expression through chromatin remodeling pathways [25,26]. Among HER family, the expression of EGFR, HER2 and HER3 have been observed in a substantial percentage of type II endometrial cancer patients and generally correlates with a shorter disease-free survival [205,206,207,208,209,210,211]. Since HER family members transduce their mitogenic and invasive responses via combinatorial heterodimerization of HERs upon the activation of ligands to EGFR or HER3, cancer cells could manifest a full repertoire of HER signaling without co-overexpression of HER family members. Similar to breast cancer, endometrial cancer shows an inverse relationship between the levels of HER2 and ERα [212].

### 7.2. DNA Methylation and Gene Expression

One of the most characterized epigenetic modifications in endometrial cancer is hypermethylation of regulatory genes during endometrial oncogenesis. It is generally accepted that differences in the pathobiology of early-onset versus late-onset endometrioid endometrial cancer might be due to differential DNA methylation of target genes belonging to mismatch repair (MMR) pathway, signaling and transcription pathways such as *Wnt*, *FGF, HOX* etc. [200]. In one such study, the status of methylated loci onto the promoters of tumor suppressor genes (i.e., *PTEN, hMLH1, CDH1* and *APC*) progressively increases from endometrial to complex hyperplasia [213]. As MMRs participate in efficient repair of mismatched bases, any potential misregulation, mutation, or silencing of *MMR* genes—as is the case in about 40% of endometrial cancer—could compromise MMR’s repair activity, and in-turn, contribute to microsatellite instability of tumor suppressor genes such as *PTEN* [199]. Interestingly, this study suggest that MMR methylation and its functional dysregulation could be recognized several years before the manifestation of carcinoma phenotypes [213], highlighting the significance of DNA methylation in prognosis of endometrial cancer. In addition to MMR silencing and associated microsatellite instability, USC also exhibits hyperactivation of c-Myc oncogene due to its gain-of-function mutations [213,214,215], and this could hyperstimulate the growth promoting signals. 

### 7.3. Emerging Role of DNA 5-mC Demethylation 

The levels of TET1 and TET2 (but not TET3) have been shown to be reduced in endometrial cancer as compared to matching normal [216], and reduced levels of TET1/2 correlate with reduced levels of 5-hmC and increased tumor aggressiveness (Figure 6b). As obesity and insulin resistance are commonly found in patients with endometrial cancer [8,9], a recent report suggests a role of increased non-genomic GPER signaling in endometrial cancer cells through TET1 upregulation in tumor microenvironment (Figure 6e), through a yet, to-be defined mechanism [217]. Significance of this work resides in the fact it connect TME with nongenomic signaling via an epigenetic pathway. 

The levels of infiltrating tumor-associated macrophages (CD68+ and CD163+ cells) positively correlate well with aggressive endometrial cancer as infiltration of such cells progressively increases from normal endometrium to hyperplastic to endometrial cancer [218]. The study also found that macrophage-derived cytokine IL17A stimulates the expression of ERα in tumor cells through relieving the methylation-linked inactivation of ERα via upregulating the expression of TET1 (Figure 6f). In addition, endogenous levels of TET1 appears to influence responsiveness of endometrial cancer cells to progestin as Metformin has been shown to downregulate TET1’s expression as well as sensitize endometrial cancer cells to progestin [219]. These observations highlight the role of functionally relevant interactions between the endometrial tumor cells and tumor microenvironment in estrogenic responses (Figure 6a). 

### 7.4. Histone Acetylation and Chromatin Remodeling

The components of chromatin remodeling pathways are integral part of epigenetic regulation of oncogenesis and are widely dysregulated in human cancer, including in endometrial cancer [25,26]. For example, mutations in the chromo, ATPase and Helicase domains in chromodomain 4 (CHD4)—a component of the NuRD/Mi2 complex, in ARID1A encoding BAF250a—a component of SWI/SNF remodeling complexes, and missense mutations in histone H3-lysine-4 methyltransferase MLL3 are frequently found in endometrial cancer [220]. As many in-frame ARID1A mutations impair its translocation to the cytoplasm and promotes its degradation by the proteasome, the loss of ARID1A tumor suppressor could also support oncogenesis [221,222]. Interestingly, an ARID1A homolog, ARID1B has an opposing function to that of ARID1A and leads to hyper proliferation of cells with mutant ARID1A. As components of both PI3K and AKT pathways are commonly stimulated in endometrial cells with mutant ARID1A, such cells have been shown to be hypersensitive to therapeutic inhibition of the PI3K/AKT pathway [223,224]; similarly, cells with mutant *ARID1A* could leads to increased EZH2 methyltransferase activity and thus, the growth of such cells could be inhibited by EZH2 inhibitors [225,226]. There are also examples of activation of SOX4 oncogene in endometrial cancer by its de-repression from an inhibitory control of microRNA-122-2 triggered by its epigenetic repression [227]. 

Like many other genes, the status of chromatin remodeling components directly influences the expression of PR-B. For example, inhibition of MBD-associated hypermethylation of *PR-B* leads to increased H3/H4 acetylation and reduced H3-K9 methylation onto the PR-B gene chromatin [228]. The expression and biology of PR-B are also regulated by epigenetic control of *PR-B* transcription by non-coding RNAs (lncRNA) such as HOX transcript antisense intergenic RNA (HOTAIR) which is widely upregulated in endometrial cancer [229] and implicated in estrogen-signaling associated invasion of endometrial cancer cells [230]. Emerging data suggest that HOTAIR, in cooperating with LSD1, suppresses the levels of PR-B expression and confers resistance to progesterone therapy in endometrial cancer cells, while the HOTAIR knockdown leads to increased deposition of H3K4me2 marks onto PR-B chromatin [231]. These representative examples illustrate how specific defects in multiple components of the epigenetic machinery, in-conjunction of other commonly found mutations in endometrial cancer cells, could lead to hyperstimulation of downstream effector molecules. This in-turn, offers an opportunity for selecting sub-sets of patients with endometrial cancer on the basis of a battery of epigenetic endpoints for exploratory combination therapies targeting such core hyperactivated pathways.

## 8. Cervical Cancer

**Figure 7:**
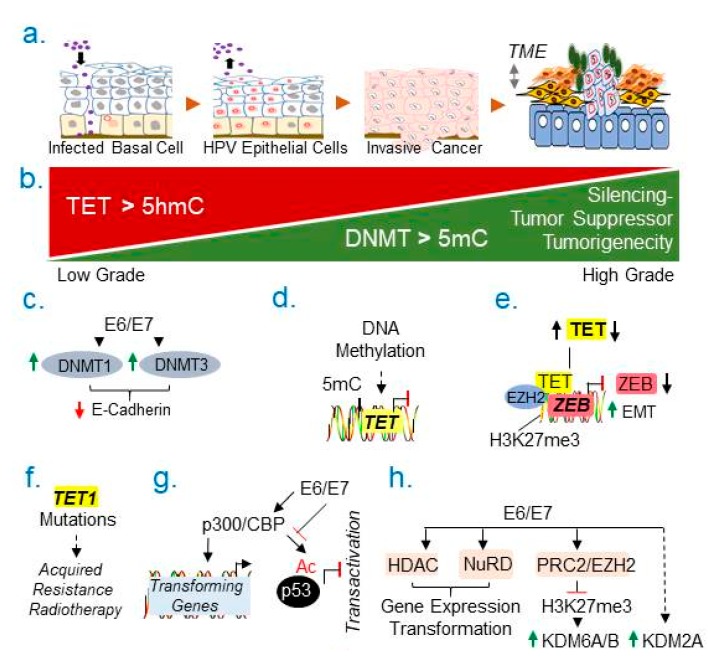
Epigenetic regulation of cervical cancer development. (**a**) Illustrations showing HPV-driven cervical cancer development and interaction with tumor microenvironment (TME). See Supplementary Figure S1 for description of TME. (**b**) Reduced levels of TETs and 5hmC and increased levels of DNMT and 5mC in cervical cancer. (**c**) Regulation of DNMT expression by E6/E7 oncoproteins. (**d**) Regulation of TET expression by DNA methylation pathway. (**e**) TET regulation of EMT via modifying the expression of EMT master regulator ZEB1. (**f**) Potential role of TET1 mutations in acquired resistance to radiotherapy. (**g**) E6/E7 regulation of target gene expression via modifying the activity of HATs and p53 acetylation. (**h**) E6/E7 regulation of gene expression via HDACs, NuRD complexes, and PRC2/EZH2 complexes.

Among women’s cancer, cervical cancer is third most common cancer globally. Stable integration of human papillomaviruses (HPVs) and transforming activities of HPV encoded oncoproteins are widely accepted major cause for the development of cervical cancer [232,233]. Pathobiology of cervical epithelium—HPV infection and its transition towards a multistep carcinogenesis suggests that about 80–90% of HPV-infected cells are cleared in about two years, while just only percentage, but not all, of cases of HPV-infected cells transition into cervical cancer over time [232,233], while the fate of remaining HPV-infected cases remains not well understood (Figure 7a). 

We do not precisely know the basis of natural selection of only a small percentage of HPV-infected cells from a pool of remaining non-cleared HPV-infected cells. This might be one of the major gaps in the field. It remains possible that cellular epigenetic pathways and their upstream regulation by extracellular and environmental signals might constitute the missing link and somehow, affect only a very small percentage of infected cells, allowing a full manifestation of their phenotypic outcome. Though this hypothesis reinforces the significance of epigenetics in pathobiology of cervical cancer, this need to wait for experimental validation. Like other female cancer, cervical cancer cells and cancer specimens exhibit dysregulated epigenetic pathways, leading to upregulation of oncogenes, silencing of tumor suppressors, and misregulation of other genes with roles in pathogenesis of the disease [234,235,236]. 

The process of multi-step transitioning of the normal cervical epithelium to precancerous cervical intraepithelial neoplasia (CIN) to invasive stages is influenced by HPV-encoded gene products and by epigenetic switches targeted by HPV-coded E6 and E7 [234]. Transforming activity of HPV is driven by the ability of E6 and E7 oncoproteins to inactivate tumor suppressors as well as modulate the interaction, activity, and functions of cellular epigenetic regulators. These regulatory changes ultimately lead to dysregulation of target genes with functions in oncogenesis. Epigenetic mechanisms contribute to the expression of both host and HPV genes in cervical epithelium cells. For epigenetic regulation of HPV genome in infected cervical epithelial cells, we refer the readers to other excellent reviews in the field [234,235,236]. 

### 8.1. DNA Methylation and Gene Expression

DNA methylation of CpG islands is one of the most prominent epigenetic modifications wherein both hyper- and hypo-methylation could have decisive contribution to the progression of cervical cancer [237,238,239,240]. For a productive modifying effect on gene transcription, the effect of DNA methylation varies in the context of chromatin status and could be methylation-dependent or -independent. Examples of CpG hypermethylation targets in cervical cancer cells include, silencing of tumor suppressor BRCA1, DNA-repair enzyme MGMT, mismatch repair enzymes (MLHs) etc. The progression of cervical cancer to more invasive phenotypes is also driven by hyperstimulation of WNT signaling due to hyper-methylation linked repression of a secreted WNT antagonist DICKKOPF-1 in cervical carcinoma cell lines [241]. Similarly, *PTEN* has been shown to be hypermethylated in cervical cancer [242], implying a role of hyperstimulation of PI3-Kinase/AKT signaling axis in cervical cancer. 

Components of DNA methylation machinery are also targeted by HPV oncoproteins in cervical cancer cells or tissues. For example, HPV-E7 is known to regulate the expression, binding, and activity of DNMT1 [243,244,245]. In general, increased levels of DNMT1 in cervical tumors have been linked with a poor outcome [246], due to silencing of tumor suppressor genes. For example, E6 and E7 proteins stimulate the expression of DNMT1 via pRB and p53 pathways, respectively [243,244,245]. Interestingly, E7 binds to DNMT1 in a manner that positively regulates its methyltransferase activity (Figure 7c). HPV E6/E7 oncoproteins also repress the expression of E-cadherin and cell-cell adhesion, leading to invasive phenotypes of cancer cells [247,248]. Similarly, other methytransferase family members, such as DNMT3B, are involved in invasive cervical cancer cells as well as DNMT3B-mediated silencing of the protein tyrosine phosphatase receptor type R [249]. In-principle, these observations support the rationale of validating the status of above and other endpoints relevant to DNA-methylation in human HPV-positive and -negative cervical cancer specimens. Future results from such translational studies might strengthen the prospect of using DNMT inhibitors in cervical cancer. 

As patients with cervical cancer are resistant to most of commonly used cytotoxic alkylating treatment modalities, cancer cells from such patients generally have defective DNA repair components such as the Fanconi Anemia (FA)-BRCA pathway. For example, *FANCF* gene is hypermethylated and repressed in cervical cancer cells [250]. It is possible that strategies to re-express *FANCF,* might increase the sensitivity of such patients to standard treatment options for invasive cervical cancer. 

### 8.2. Emerging Role of DNA 5mC Demethylation

Like other cancer, the expression of TET1 is also reduced in cervical cancer [251], implying, an inherent role of TET activity in maintaining the hypermethylated status of target genes, and consequently, silencing of target genes. Interestingly, TET1 itself appears to be also a target of DNA methylation epigenetic silencing (Figure 7d), in cervical and breast cancers [252,253]. The levels of TET1 and its enzymatic hydroxylated product, the 5hmC, are shown to be upregulated in the normal cervix as compared to invasive stage (Figure 7b). In experimental models, TET1-depletion leads to increased EMT and repression of *ZEB1* via physically interacting with LSD1 and EZH2 on the *ZEB1* promoter (Figure 7e), through 5hmC-dependent or -chromatin remodeling dependent-manner [253]. There are also examples of TET1 mutations in uterine cervical cancer with acquired resistance to radiotherapy (Figure 7f) [254], raising the possibility of adding TET1 to the growing list of molecules implicated in acquired therapeutic resistance. 

### 8.3. Histone Acetylation and Chromatin Remodeling 

HPV E6 and E7 oncoproteins modulate the expression of cellular genes involved in cancer progression via acetylation of histones or non-histones, and also affecting the interaction and activity of components of the chromatin remodeling machinery (Figure 7g). For example, E6 and E7 oncoproteins target HATs, such as p300 and CBP, to regulate a set of transformation genes [255,256,257,258]. E7 oncoprotein acetylates pRB via interacting with p300/CBP [259] as well as disrupting pRB’s cell cycle function [260]. E6 protein also inhibits p300/CBP-mediated acetylation of p53 acetylation and its transcriptional activity in a manner which was independent of its p53 degradation [261,262]. 

In addition to HATs, E7 oncoprotein also interact with HDACs and the Mi2β, a component of the nucleosome remodeling and histone deactylation (NuRD) complex with role in nucleosome repositioning onto target gene chromatin (Figure 7h). It appears that the interaction of E7 with HDAC or Mi2β may have a functional ramification as a point mutation in the E7 zinc-finger domain impairs its ability to bind to Mi2β and HDAC1 and abolishes its transforming activity [263,264]. Another example of HPV-oncoprotein targeting chromatin remodeling pathways includes the polycomb repressive complexes (PRC) which are responsive for repressive H3K27me3 modification and supressing the expression of target genes [265]. In general, E7 oncoprotein inhibits H3K27me3 repressive mark in HPV16-infected cells [266]. The PRC2 complex which contains methyltransferase EZH2 is known to be dysregulated in E7-infected cervical cancer cells [267]. As components of PI3K/AKT pathways are widely mutated and hypersactivated in human cancer and because E6/E7 oncoproteins stimulate the AKT signaling, AKT phosphorylation of EZH2 inhibits its activity [268,269,270], contributing to the loss of corepressive activity of EZH2 and in-turn, de-repression of cancer relevant genes. Further, inhibition of H3K27me3 repressive modification could be also related to increased expression of lysine demethylases, KDM6A and KDM6B, in cervical cancer cells [271]. There are also examples of E7-regulated increased expression of KDM2A during the progression of cervical cancer to aggressive stage. In addition to lysine methyltransferases and demethylases, E6 oncoprotein inhibit the activity of histone arginine methyltransferases in HeLa cells and contribute to suppression of p53 transactivation function [272,273]. 

Acetylated lysine is recognized by the bromodomain containing BET family chromatin-binding proteins which have a fundamental role in transcription elongation by RNA polymerase II. One of the most characterized BET family members, the Brd4, recognizes the target genes via interacting with methylated histones H3 or H4 [274]. HVP E2 protein interacts with Brd4 thoughts its C-terminal domain, inhibits its ability to interact with pTEFb, and in-turn, suppress the expression of E6/E7 [275,276]. This suggests that HVP encoded genes might be also trans-regulated by cellular proteins. 

In brief, these examples of HPV-modulation of enzymes and/or complexes with roles in adding or removing the acetylation marks in histone and non-histone regulatory proteins are expected to have wide-ranging effects on the expression of cellular genes. As many of these and other mechanistic studies of E6/E7 oncoproteins have not always used cervical cancer cells, this is an understudied area in cervical cancer research and waiting to unearth a full repertoire of target cancer genes and their underlying regulatory mechanisms.

## 9. RNA Methylation in Women’s Cancer

Similar to DNA, chemical features of mRNAs, and hence, resulting functions, are modified by reversible posttranslational modifications. In recent years, this area of research has introduced a few new facets in the regulation of gene expression and emerged as ‘RNA epitranscriptomics’ [40,41,42]. Here we will high light the role and dysregulation of enzymes that regulate adenosine methylation in mRNA at position 6, *N*^6^-methyladenosine—one of the most abundant internal RNA modifications—close to the 3’-UTR within the consensus motif GAC or AAC in human cancer cells with a particular focus on female cancer. 

Dysregulated levels of m6A metabolism and/or enzymes responsible for adding, recognizing and removing this PTM by writer, reader and eraser proteins could have growth-promoting or -inhibitory functions in a context-dependent manner. The m^6^A modification is carried by writer proteins such as methyltransferases such as METTL3 and METTL14, and an auxiliary factor Wilms’ tumor 1-associated protein (WTAP); while eraser proteins such as demethylases such as FTO, ALKBH1 and ALKBH5 demethylate the methylated m^6^A [277,278,279,280]. The modified CH3 moiety on m^6^A is recognized by reader proteins such as YTHDC1 and YTHDF1-3 (Figure 4b). Many of such reader proteins have a fundamental role in mRNA biology. For example, YTHDC1 is important for the process of splicing, mRNA export to the cytoplasm, mRNA stability, and mRNA translation [279,280,281]. From a very limited number of studies in women’s cancer as a whole, it’s clear that the levels and activities of m^6^A-interacting proteins and enzymes are dysregulated in female cancer, and introduce a new regulatory layer to transcriptomics. A defective regulation of any of these steps is expected to impact the biology of mRNAs of target cancer relevant genes. 

### 9.1. Breast Cancer

**Figure 8:**
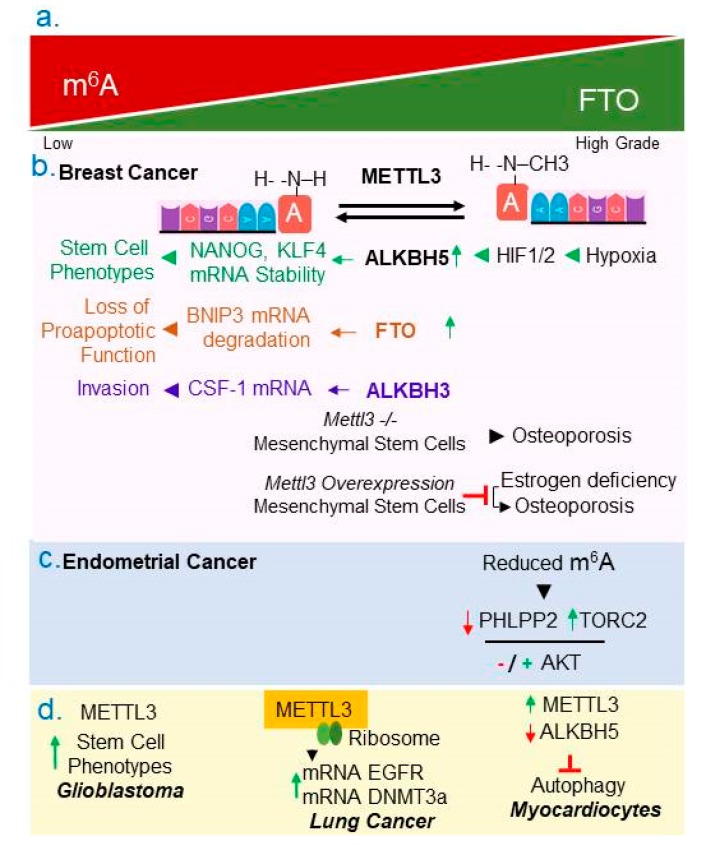
RNA m^6^A methylation and women’s cancer. (**a**) Illustrations showing a progressive loss of m6A modification and increase FTO status during cancer progression. (**b**) Effect of increased expression of exemplified RNA-demethylating enzymes in modifying the stability of indicated target mRNAs and resulting functions in breast cancer cells. Also shown is the effect of conditional *Mettl3* depletion or overexpression in mesenchymal stem cells on osteoporosis in mice. (**c**). Effect of reduced m^6^A expression on coordinated regulation of negative and positive regulators of AKT signaling in endometrial cancer cells. (**d**) Examples of modifying effects of METTL3 levels in glioblastoma cancer cells, lung cancer cells, and myocardiocytes. Status of these cellular effects of METTL3 in women’s cancer remains unknown.

In general, expression of m^6^A methyltransferases is downregulated in breast cancer when compared with normal tissues (Figure 8a). Interesting, either METTL3 overexpression or ALKBH5 depletion in breast cancer cells leads to suppression of cancerous phenotypes [282]. The status of m^6^A RNA methylation has been shown to modulate stem cell phenotypes in breast cancer cell under hypoxic environment [283,284]. The underlying mechanism involves HIF1α- and HIP2α-dependent increased expression of ALKBH5 demethylase, leading to demethylation of NAOG mRNA and an enhance NAOG mRNA expression and breast cancer stem cell phenotypes (Figure 8a). Further, m^6^A demethylase FTO has been also found to be overexpressed in breast cancer and associates with a poor survival of breast cancer patients [285]. Significance of FTO in breast cancer is also evident by its positive role for supporting the growth of breast cancer cells in anchorage-independence and metastasis models [285]. Mechanistically, increased expression of FTO could leads to m^6^A demethylation and degradation of proapoptotic BNIP3 mRNA (Figure 8a). In addition to m^6^A, methylation of m^1^A also regulates mRNA functions. In this context, a recent study suggested that m^1^A demethylase ALKBH3 enhances the stability of CSF1 mRNA in breast and ovarian cancer cells and hence, promotes its expression as well as invasion of cancer cells (Figure 8a). Increased expression of CSF1 is known to be associated with a poor prognosis [286]. Expression of METTL3 has been also shown to be positively regulated by hepatitis B X-interacting protein (HBXIP) by downregulating the levels of let-7g during breast cancer progression [287].

One of the major complications of hormonal therapy of breast cancer is osteoporosis—a process associated with a reduced systemic level of estrogen (and by implication, ERα in signaling) [288,289]. At the moment, there is no report to connect these physiologically relevant processes of estrogenic signaling with RNA-methylation in women’s cancer. In this context, a recent study suggests that experimental depletion of *Mettl3* in mesenchymal stem cells could lead to the development of osteoporosis in a mice model [290], while its upregulation provides protection against osteoporosis triggered by estrogen deficiency. These authors found that m^6^A targets parathyroid hormone receptor-1 mRNA and that *Mettl3* depletion impairs Pth1r mRNA translation [290], providing clues about the role of RNA-methylation on estrogen signaling in women’s cancer.

### 9.2. Endometrial Cancer

RNA methylation is a new area of research in endometrial cancer. RNA methytransferase METTL14 contains a hotspot mutation at R298P in endometrial cancer [291,292]. In this context, endometrial tumors has been shown to exhibit a decreased level of m^6^A methylation as well as reduced expression PHLPP2 and increased expression of TORC2—negative and positive regulators of AKT signaling, respectively (Figure 8b) [292]. This suggests that reduced expression of METTL14 or its mutational inactivation could lead to increased AKT signaling. 

### 9.3. Cervical Cancer

We do not know the expression and significance of methylated m^6^A in cervical cancer. From very limited information, the levels of methylated m^6^A mRNA appears to be reduced in cervical cancer and associates with cancer progression and disease-free survival of patients with cervical cancer [293].

### 9.4. Outstanding Questions about the Role of RNA-Methylation in Female Cancer

There are several recent observations in other systems that might be worth examining in female cancer. Examples of such findings include: association of increased expression of methyltransferases METTL3 and METTL14 with the self-renewal of glioblastoma stem cells via influencing the expression of putative target mRNAs of m^6^A, such as ADAM19 (Figure 8c) [294]; METTL3 interaction with ribosomes and assistance in translation of target mRNAs [295]; and expression and functionally relevant mutations in METTL3 could influence mRNA translation in the cytoplasm (Figure 8c). Interestingly, increased expression of METTL3 in lung cancer cells leads to increased translation of growth factor receptor EGFR as well as a epigenetic modifier DNMT3a [295]. This raises new mechanistic possibilities for women’s cancer, including, status of this pathway in women’s cancer; role of RNA-epigenetics on DNA-methylation via DNMT3a; role on the expression and feedback regulatory roles of HER2, HER3, HER4, ER and PR; and role of RNA-epigenetics on DNA-methylation via DNMT3a in breast cancer. 

Because autophagy plays an important role in the development of mammary gland and breast cancer and therapeutic resistance [296], the significance of RNA-methylation in autophagy in female cancer remains unknown. In this context, METTL3 overexpression as well as suppression of ALKBH5 in cardiomyocytes leads to inhibition of autophagy [297]. Mechanistically, METTL3 targets autophagy genes and that methylated genes promote METTL3’s interaction with the RNA-binding protein, suggesting a previously unknown role of RNA-methylation pathway, i.e., METTL3 and ALKBH, in autophagy. Significance of these METTL3-regulated phenotypes in women’s cancer remains unknown.

## 10. Clinical Outlook 

It is clear that epigenetic anticancer therapies hold a great promise both as biomarkers and therapeutic targets, as evidenced from a large number of early stage clinical studies, proof-of-concept preclinical data, and new discoveries and leads from laboratory model systems. Examples of such advances include: DNMT-, HDAC-, BET- and KDM-inhibitors, as well as reagents to assay the status of epigenetic PTMs in histones and non-histone proteins. As these themes are discussed in several excellent reviews [298,299,300], the authors have chosen to briefly highlight in this publication examples of some of the most recent advances in epigenetic regulation of acquired resistance and examples of continuing limitations. 

One of the major bottlenecks in breast cancer treatment is acquired resistance to endocrine- and HER-directed therapies. At the moment, there are no epigenetic-centered therapies or prognostic panels to predict which patients are likely to develop therapeutic resistance. However, there are some potentially important leads which could be further investigated. For example, as histone demethylase KDM5, which removes tri- and di-methyl marks from histone H3 lysine 4, participates in acquired endocrine resistance in breast cancer, it might be possible to target KDM5 to restore ER signaling and sensitivity to anti-estrogen therapy [301]. 

In addition, KDM5-inhibitors have been shown to act synergistically with trastuzumab and lapatinib—two commonly used drugs to treat HER2+ positive breast cancer, in HER2+ breast cancer cells [302], and also induce cell-cycle arrest and senescence in a battery of breast and other cell lines [303]. As these studies were largely transcriptomic centered, the nature of the molecular basis for the noted synergistic activity remains undefined [303]. Interestingly, the authors of the referred study noted that the observed synergistic activity of KDM5 inhibitors with trastuzumab and lapatinib—two commonly used drugs to treat HER2+ positive breast cancer—was independent of its target, the KDM5 [303]. This re-enforces the notion of possible off-target beneficial effects of epigenetic inhibitors and emphasizes the need to also start developing strategies to understand this phenomenon. Results from transcriptomic and proteomic analysis of breast cancer patients which were either sensitive or resistant to the aromatase inhibitor letrozole revealed a substantial reduction in the levels of DNA 5-methylcytosine and 5-hydroxymethylcytosine in resistant tumors [304]. Similarly, proof-of-concept studies using TNBC cell lines with differential sensitivity to BET bromodomain protein BRD4 inhibitors revealed a correlation between the MED1 status and BRD4 hyperphosphorylation in resistant cells [305]. Because these representative and many other studies using experimental models reveal the significance of a given molecule/pathway individually, the challenge in the field is to continue to evaluate these molecules in the context of multiple pathways converging together—as is the case in patients where these molecules are not working in isolation. Because a full-blown acquired therapeutic resistance is developed over a period of time, the authors anticipate that unless we have an integrated portrait of epigenetic nodules in sync with transcriptomic changes and resulting proteins and their functionality, it might be difficult to achieve the goal of a stable reversal of therapeutic resistance or substantially delay the development of acquired resistance to a point which allows eradication of tumors by designer molecules. These are some of the areas where the authors are formulating testable hypotheses.

## Supplementary Materials

The following are available online at https://www.mdpi.com/2072-6694/11/8/1193/s1, Figure S1: Representative components of the tumor microenvironment (TME) illustrated in Figure 5a, Figure 6a, and Figure 7a.
