# Supplementary material for: Epigenetic Dysregulation at the Crossroad of Women’s Cancer
Source: Cancers (Basel). 2019 Aug 16;11(8):1193. doi: 10.3390/cancers11081193 (PMC6721458; doi:10.3390/cancers11081193)
Supplement: Supplementary file 1 [file cancers-11-01193-s001.pdf]

cancers

Supplementary Material

Epigenetic Dysregulation at the Crossroad of Women's Cancer

Rakesh Kumar, Aswathy Mary Paul, Pranela Rameshwar and M. Radhakrishna Pillai

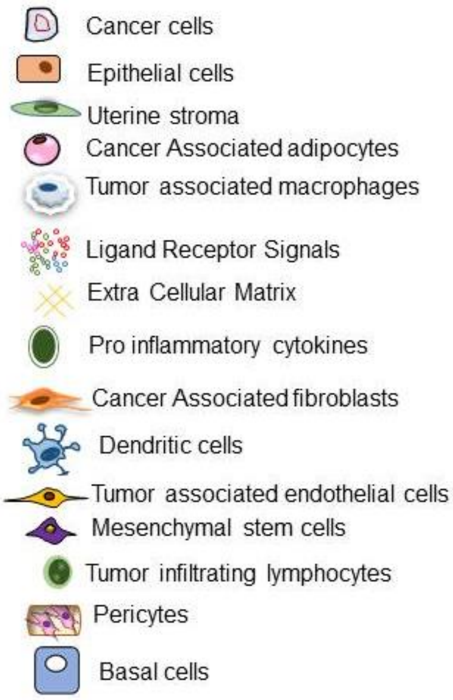

Figure S1. Representative components of the tumor microenvironment. (TME) illustrated in Figures 5a, 6a, and 7a.
